# Nitrogen-Doped Carbon Nanoparticles for Oxygen Reduction Prepared via a Crushing Method Involving a High Shear Mixer

**DOI:** 10.3390/ma10091030

**Published:** 2017-09-04

**Authors:** Lei Shi, Tao Wu, Yiqing Wang, Jie Zhang, Gang Wang, Jinli Zhang, Bin Dai, Feng Yu

**Affiliations:** 1 Key Laboratory for Green Processing of Chemical Engineering of Xinjiang Bingtuan, School of Chemistry and Chemical Engineering, Shihezi University, Shihezi 832003, China; shilei70058@163.com (L.S.); wutao258@foxmail.com (T.W.); wangyiqing_yes@sina.com (Y.W.); Zhangjie@shzu.edu.cn (J.Z.); wanggang@shzu.edu.cn (G.W.); zhangjinli@tju.edu.cn (J.Z.); db_tea@shzu.edu.cn (B.D.); 2 Key Laboratory of Materials-Oriented Chemical Engineering of Xinjiang Uygur Autonomous Region, Shihezi 832003, China; 3 Key Laboratory of Xinjiang Phytomedicine Resources of Ministry of Education, School of Pharmacy, Shihezi University, Shihezi 832002, China

**Keywords:** agricultural wastes, porous carbon nanoparticles, fresh banana peel, high shear mixer, oxygen reduction, energy conversion

## Abstract

The disposal of agricultural wastes such as fresh banana peels (BPs) is an environmental issue. In this work, fresh BPs were successfully transformed into nitrogen-doped carbon nanoparticles (N-CNPs) by using a high shear mixer facilitated crushing method (HSM-FCM) followed by carbonization under Ar atmosphere. Ammonia-activated N-CNPs (N-CNPs-NH_3_) were prepared via subsequent ammonia activation treatments at a high temperature. The as-prepared N-CNPs and N-CNPs-NH_3_ materials both exhibited high surface areas (above 700 m^2^/g) and mean particle size of 50 nm. N-CNPs-NH_3_ showed a relatively higher content of pyridinic and graphitic N compared to N-CNPs. In alkaline media, N-CNPs-NH_3_ showed superior performances as an oxygen reduction reaction (ORR) catalyst (E_0_ = −0.033 V, J = 2.4 mA/cm^2^) compared to N-CNPs (E_0_ = 0.07 V, J = 1.8 mA/cm^2^). In addition, N-CNPs-NH_3_ showed greater oxygen reduction stability and superior methanol crossover avoidance than a conventional Pt/C catalyst. This study provides a novel, simple, and scalable approach to valorize biomass wastes by synthesizing highly efficient electrochemical ORR catalysts.

## 1. Introduction

Bananas are one of most consumed fruits in the world, and the worldwide production reaches 100 million tons per year. Significant amounts of organic banana peel (BP) wastes up to more than 30 million tons are produced every year [1]. BPs accounts for 1/3 of the bananas total weight and, together with rice husks and sugarcane bagasse, they are one of the largest agricultural residues. This large amount of BP wastes causes pollution of the environment, and a method to valorize this agricultural residue is urgently needed. In this sense, BPs can serve as a source of carbon-based materials that can be used as electrodes for a large number of applications [2].

Biomass-derived carbon materials have received a great deal of attention. Among these, pomelo peel [3], catkin [4],coconut shell [5], tea leaves [6], corn husk [7], cotton stalk [8], and others [9,10], have been used to prepare carbon materials for energy storage applications. BP-derived carbons have been employed as environmental adsorption materials [11] and supercapacitors [12], among other applications [13]. Remarkably, all the above mentioned processes involve a natural or apparatus-based drying step. The storage and drying of fresh BPs still remain challenging.

Fuel cells are regarded as a renewable energy technology and have been widely studued in recent years for their high energy density and zero emission [14]. However, advance in fuel cells is limited due to the sluggishness of the oxygen reduction reaction (ORR) in the cathode. Traditionally, platinum and its alloys are the most effective ORR catalysts. However, the main drawbacks of Pt or Pt-based electrocatalysts including high price, intolerance to methanol, and instability in the fuel-cell environment have greatly limited their use in energy storage devices [15,16,17]. In order to meet the economic requirements of fuel cells technologies, inexpensive and commercially available [18]. Heteroatom-doped porous carbon materials have been proven to be ORR catalysts in recent studies [13,19,20,21]. Zhou et al. developed microorganism-derived heteroatom-doped carbon materials from bacillus subtilis for oxygen reduction. The obtained sample exhibited excellent electrocatalytic activity, long-term stability, and excellent resistance to crossover effects in oxygen reduction [22]. Yuan et al. reported that nitrogen-doped nanoporous carbon derived from waste pomelo peel can be regarded as metal-free electrocatalyst for the oxygen reduction reaction [23]. Wang et al. used sterculia scaphigera to produce ultrafine N-doped carbon nanoparticles with controllable size to enhance the electrocatalytic activity for the oxygen reduction reaction [24]. Li et al. reported that nitrogen-doped graphitic porous carbons derived from in situ formed g-C_3_N_4_ templates for the oxygen reduction reaction and show excellent electrochemical performances [25].

Herein, we successfully developed a drying-free approach to crush fresh BPs into a BP precursor emulsion via a high shear mixer facilitated crushing method (HSM-FCM). The BP-precursor emulsion was subsequently transformed into nitrogen-doped BP-derived carbon nanoparticles (N-CNPs) via a hydrothermal reaction followed by high temperature carbonization. Compared to other processing routes used for the prepreparation of nanostructured carbon materials, the HSM-FCM method developed herein has the following advantages: (i) BPs can be directly crushed without the need for a natural or apparatus-based drying step; (ii) a high shear stress based technology can be used to directly crush BPs into an emulsion. High-shear mixers (HSMs) are widely utilized to prepare fine dispersions and to carry out uniform reactive mixing processes owing to their high mass and heat transfer rates as compared to conventional mechanical impellers [26]; (iii) the high-shear process results in BPs having a spherical shape after the pyrolysis step. These materials can be subsequently used as oxygen reduction reaction catalysts for fuel cell applications owing to their environmental friendliness and excellent energy conversion efficiency. The main challenge in research on fuel cells lies in the development of low cost and highly efficient cathode electrocatalysts for the sluggish ORR [16,27]. Additionally, the HSM-FCM method developed herein provides a strategy for the rapid treatment of fresh BPs, which could be potentially extended to other agricultural waste materials.

## 2. Results and Discussion

### 2.1. Morphological Characterization

**Figure 1:**
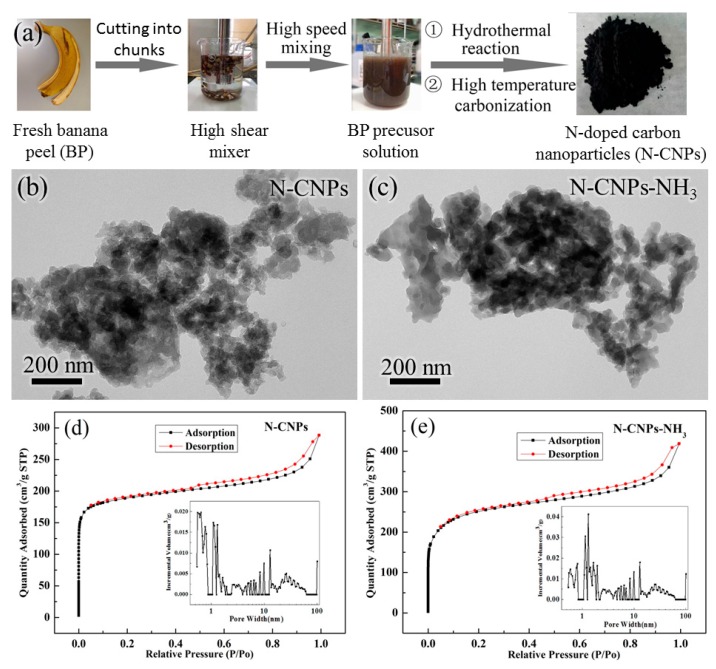
(**a**) Description of process from fresh banana peels to porous carbon. TEM images of (**b**) N-CNPs and (**c**) N-CNPs-NH_3_. Nitrogen sorption isotherms and the corresponding pore size distributions of (**d**) N-CNPs and (**e**) N-CNPs-NH_3_.

The preparation methodology of N-CNPs is schematically summarized in Figure 1a. Fresh BPs were directly crushed into an emulsion via a drying-free HSM-FCM. The high mass and heat transfer rates of the HSM-FCM as compared to conventional mechanical impellers allowed the facile synthesis of N-CNPs after hydrothermal carbonization, as revealed by transmission electron microscopy (TEM) imaging (Figure 1b). The TEM image in Figure 1c shows that the black N-CNPs-NH_3_ material obtained after nitrogen doping via ammonia activation has similar morphology to that of N-CNPs. Thus, we can conclude that the morphology of N-CNPs remained unchanged after ammonia activation. As observed by TEM (Figure 1b,c), both N-CNPs and N-CNPs-NH_3_ materials possessed a significant amount of holes. These channels, in good contact with the particle surface, can serve as continuous electron pathways, thereby potentially improving the stability of the materials.

In order to further verify the effects of these channels, N_2_ adsorption–desorption isotherms were generated to obtain the pore size distribution and specific surface area (SSA) of N-CNPs and N-CNPs-NH_3_ samples (Figure 1d,e). As shown in Table 1, both N-CNPs and N-CNPs-NH_3_ showed high SSA (734.8 and 941.2 m^2^/g) with pore size distributions centered at ca. 2.4 and 2.7 nm, respectively. N-CNPs-NH_3_ showed higher SSA and higher porosity than N-CNPs as a result of the ammonia activation treatment. More importantly, when compared to N-CNPs, N-CNPs-NH_3_ contained a larger number of edges and defects, whose presence is important to improve the ORR activity. The hysteresis loop of the N_2_ adsorption–desorption isotherms (Figure 1d,e, type-H4 isotherms) was indicative of the existence of micro- and mesopores, which agreed with the pore size distribution (inset) and TEM results.

### 2.2. Structure and Composition Characterization

**Figure 2:**
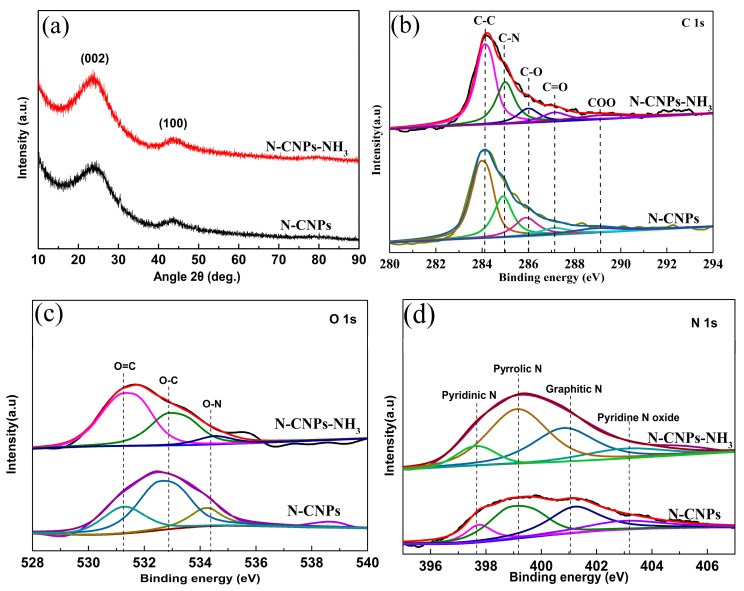
(**a**) XRD patterns of the N-CNPs and N-CNPs-NH_3_ samples. High-resolution XPS spectra. (**b**) C 1s; (**c**) O 1s; and (**d**) N 1s bands of N-CNPs and N-CNPs-NH_3_.

**Figure 3:**
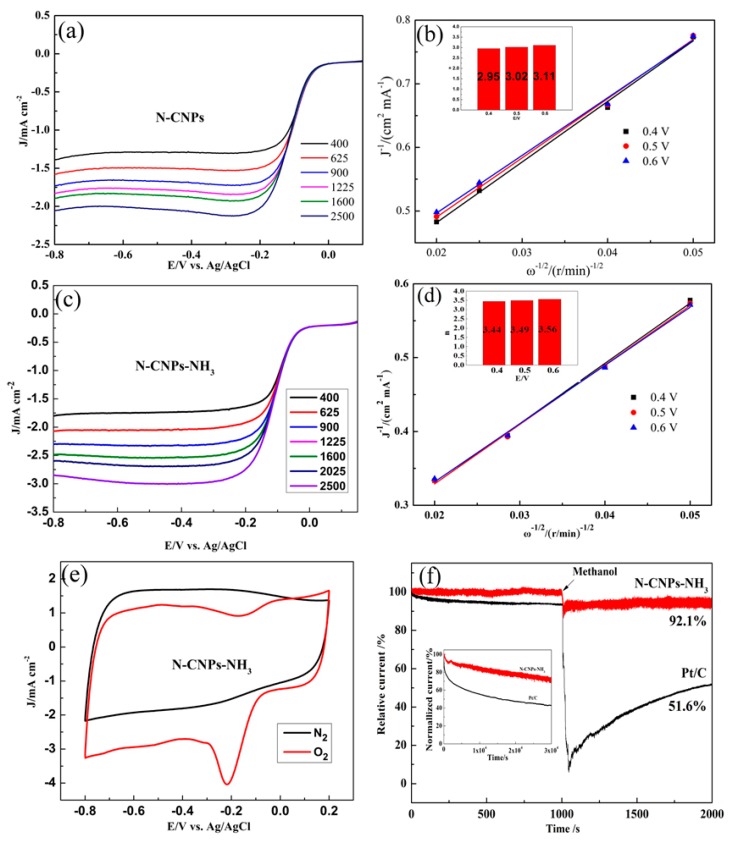
(**a**) LSV curve and (**b**) K–L plots of N-CNPs; (**c**) LSV curve and (**d**) K–L plots of N-CNPs-NH_3_. Inset: corresponding transferred electron numbers of N-CNPs and N-CNPs-NH_3_. CV curves of N-CNPs and N-CNPs-NH_3_ in 0.1 M KOH solutions at a scan rate of 50 mV/s. RDE voltammograms of: (**e**) commercial 20 wt % Pt/C, N-CNPs, and N-CNPs-NH_3_ in a O_2_-saturated 0.1 M KOH solution (rotation speed 1600 rpm); (**f**) I–t curves of N-CNPs-NH_3_ and Pt/C at 0.25 V in a O_2_-saturated 0.1 M KOH solution (400 rpm) with or without addition of a 3.0 M methanol solution. Inset: I–t curves of N-CNPs-NH_3_ with a rotation speed of 400 rpm under constant voltage (0.25 V) for ca. 30,000 s.

The X-ray diffraction (XRD) patterns of N-CNPs and N-CNPs-NH_3_ are shown in Figure 2a. All the XRD patterns exhibited the two typical broad peaks of graphite, thereby indicating the presence of an amorphous phase. In order to further understand the origin of the catalytic activity of N-CNPs and N-CNPs-NH_3_, these samples were analyzed by XPS. The C 1s spectra of both samples (Figure 2b) showed the presence of five peaks. The dominant peak at 284.2 eV can be attributed to the C–C species, while the remaining peaks at ca. 284.9, 286.0, 286.3, and 289.2 eV can be ascribed to C–N, C–O, C=O, and COO species, respectively. The C–N species mainly originated from the amino acids present in fresh BPs. Figure 3c shows the O 1s band of N-CNPs and N-CNPs-NH_3_ which was deconvoluted into three bands (i.e., O=C at 531.1 eV, O–C at 532.8 eV, and O–N at 534.4 eV), and these originated from the adsorbed oxygen atoms. The N 1s X-ray photoelectron spectroscopy (XPS) spectrum of both samples (Figure 2d) was deconvoluted into four peaks 397.6, 399.1, 401.0, and 403.2 eV corresponding to pyridinic, pyrrolic, graphitic, and pyridine N oxide species, respectively [12]. N-CNPs and N-CNPs-NH_3_ showed different pyridinic N (0.23 vs. 0.79 at %) and quaternary N (0.11 vs. 0.40 at %) contents, respectively (Table 2). N-CNPs-NH_3_ showed higher N contents than N-CNPs (2.43 vs. 1.02 at %, respectively, Table 2), thereby suggesting that a nitrogen-doped porous carbon material was successfully prepared after NH_3_ activation [1].

### 2.3. Electrochemical Consequences

In order to investigate the role of nitrogen doping and the spherical morphology of N-CNPs-NH_3_, relevant ORR electrochemical tests were carried out in N_2_/O_2_ saturated 0.1 M KOH solutions using the rotating disk electrode (RDE) method. The reaction kinetics of N-CNPs and N-CNPs-NH_3_ were evaluated via an ORR polarization method at different rotation speeds (Figure 3a,c). The Koutecký–Levich (K–L) plots of both samples (Figure 3b,d) showed a approximately linear relationships between j^−1^ and ω^−1/2^. The average values of the electron transfer number (*n*) values of N-CNPs (Figure 3b) and N-CNPs-NH_3_ (Figure 3d) were estimated from the K–L plots within the potential ranges of 0.45–0.55 and 0.4–0.6 V, respectively. The n values of N-CNPs-NH_3_ for the ORR were 3.44, 3.49, and 3.56, thereby revealing a four-electron reaction pathway (see inset of Figure 3d). In contrast, N-CNPs exhibited n values of 2.95, 3.02, and 3.11, suggesting a combination of two-electron and four-electron reaction pathways (inset of Figure 3b). The The cyclic voltammetry (CV) and linear sweep voltammetry (LSV) results allowed us to conclude that the spherical surface, nitrogen doping, and high SSA characteristics of N-CNPs-NH_3_ were responsible for the superior electrocatalytic properties of this sample [1]. To evaluate the electrocatalytic activity of N-CNPs-NH_3_, CV measurements were carried out (Figure 3e). N-CNPs-NH_3_ showed an noticeable oxygen reduction peak in O_2_-saturated 0.1 M KOH solutions, whereas this peak was not observed in the case of N_2_-saturated solutions.

As expected, N-CNPs-NH_3_ displays a better ORR onset potential of −0.033 V than N-CNPs and approaches the ORR onset potential of most biomass-derived carbon materials. The results are comparable to those reported in previous studies (Table 3), such as −0.05 V for N-doped carbon from bacillus subtilis [22], 0.01 V for N-doped nanoporous carbon from pomelo peel [23], −0.02 V for N-doped carbon nanoparticles from sterculia scaphigera [24], −0.04 V for oxygen-containing N-doped carbon materials from glucose and dicyandiamide [25].

Long-term durability is crucial for the practical application of ORR catalysts in fuel cells. The ORR stabilities of N-CNPs-NH_3_ and of a commercial 20 wt % Pt/C catalyst were compared on the basis of I–t chronoamperometric measurements (0.25 V, rotation rate: 400 rpm). As shown in the inset of Figure 3d, N-CNPs-NH_3_ preserved 70.6% of its initial relative current density after 30,000 s (vs. 43.1% for the 20 wt % Pt/C catalyst). The high catalytic stability of N-CNPs-NH_3_ may be attributed to the spherical shape of the carbon material. Methanol crossover tests were also conducted for both N-CNPs-NH_3_ and 20 wt % Pt/C samples by adding a 3 M methanol solution after 1000 s (Figure 3f). The ORR current of N-CNPs-NH_3_ only slightly decreased after methanol addition, while the 20 wt % Pt/C catalyst lost ca. 30% of its initial current within 1000 s after methanol addition.

## 3. Materials and Methods

### 3.1. Preparation of BP-Derived Carbon Materials

To obtain the BP precursor, fresh BPs (200 g) were cut into small pieces and loaded into a 500 mL beaker containing 450 mL of water. The resultant BPs–water mixture was processed on a HSM (FA25, FLUKO Equipment Shanghai Co., Ltd., Shanghai, China) at 13,000 rpm/min until complete homogenization was achieved (as revealed by the formation of a milky liquid). The milky liquid was subsequently transferred to a Teflon autoclave and hydrothermally treated at 180 °C for 12 h. The hydrothermal BP product was filtered, washed several times with HCl and deionized water, and dried at 60 °C overnight to finally obtain the BP precursor. The BP precursor was ground and pyrolyzed under Ar at 800 °C for 5 h. After grinding in an agate mortar for 20 min, a BP-derived porous carbon was obtained (i.e., N-CNPs). This N-CNPs material was subsequently activated with NH_3_ at 800 °C for 5 h to obtain the ammonia activated N-CNPs (i.e., N-CNPs-NH_3_). We herein investigated the properties of N-CNPs and N-CNPs-NH_3_ derived from fresh BPs, and well-confirmed conclusions were obtained.

### 3.2. Physical Characterization of the Samples

A Hitachi SU8010 microscope was used to perform scanning electron microscopy (SEM) imaging of the N-CNPs and N-CNPs-NH_3_ materials. TEM was carried out on a Tecnai F30 field-emission transmission electron microscope. X-ray diffraction experiments were conducted on a Bruker D8 Advance X-ray diffractometer with a Cu Kα radiation source (λ = 1.5406 Å). An AMICUS/ESCA 3400 electron spectrometer was used to collect the XPS spectra by using Mg Kα (12 kV, 20 mA) radiation. The C 1s line at 284.8 eV was used as the reference. A Micromeritics ASAP 2020 apparatus was used to analyze the Brunauer–Emmett–Teller (BET) specific surface area (SSA) and the Barrett–Joyner–Halenda (BJH) pore structure of the prepared materials at 77.35 K via the N_2_ adsorption–desorption method. Chemical mapping was conducted with a JEM-ARM 200F microscope operating at 200 kV.

### 3.3. Electrochemical Measurements

The electrochemical performances of the prepared materials were evaluated with a standard three-electrode cell using a CHI760D electrochemical workstation. A Ag/AgCl electrode was employed as a reference electrode, while a Pt foil was employed as the counter electrode. A catalyst-ink-loaded glassy carbon disk (GC, 5.0 mm in diameter) was used as the working electrode (rotating disk electrode (RDE)). The performances of N-CNPs and N-CNPs-NH_3_ were compared with those of a commercial 20 wt % Pt/C electrode. N-CNPs-NH_3_ powder (3 mg) was mixed with a Nafion solution (25 μL) and alcohol (475 μL), and the resultant mixture was ultrasonicated for 30 min. The electrochemical tests were carried out in an alkaline medium (i.e., 0.1 M KOH). Linear sweep voltammetry was carried out with the RDE in an O_2_-saturated electrolyte with a scan rate of 10 mV/s. Cyclic voltammetry was conducted in N_2_-and O_2_-saturated electrolytes at a scan rate of 50 mV/s. Additionally, the ORR stability and methanol crossover resistance characteristics were determined in an O_2_-saturated solution by performing the current versus time (i–t) test, with a revolving speed of 400 rpm and a constant potential of −0.23 V (vs. Ag/AgCl). To calculate the electron transfer number (*n*), the Koutecký–Levich (K–L) equation was used:

1/*J* = 1/*J_L_* + 1/*J_K_* = 1/*Bw*^1/2^ + 1/*J_K_*, (1)

*B* = 0.62*nFC*_0_(*D*_0_)^2/3^ν^−^^1/6^, (2)

where *J* is the current density, *J_K_* is the kinetic current density, *J_L_* is the diffusion-limited current density, *F* is the Faraday constant (96,485 C), *C*_0_ is the oxygen bulk concentration (1.2 × 10^−3^ M), *D*_0_ is the oxygen diffusion coefficient in a 0.1 M KOH electrolyte (approximately 1.9 × 10^−5^ cm^2^/s), ν is the kinetic viscosity of the solution (0.01 cm^2^/s), and ω is the electrode rotation rate.

## 4. Conclusions

N-doped carbon nanoparticles with high SSA (941.2 m^2^ g^−1^) were synthesized from fresh BPs by a facile high shear method followed by heat or ammonia activation treatments. The N-CNPs-NH_3_ demonstrated outstanding electrochemical ORR activity in alkaline media. Moreover, this material showed superior tolerance and stability in alkaline systems as a result of its relatively high pyridinic and graphitic N contents, which provide active ORR sites. In conclusion, this study provides a novel HSM-FCM approach to valorize fresh agricultural wastes and to synthesize electrochemical ORR catalysts.

## Figures and Tables

**Table 1:** Specific surface areas and pore size distribution of N-CNPs and N-CNPs-NH_3_.

Sample	S_BET_ (m^2^/g)	D_BJH_ (nm)	Pore Volume (cm^3^/g)
N-CNPs	734.8	2.4	0.23
N-CNPs-NH_3_	941.2	2.7	0.45

**Table 2:** Atomic content of N-CNPs and N-CNPs-NH_3_.

Sample	Content (%)			Content of N Species (%)			
	C (%)	N (%)	O (%)	Pyridinic	Pyrrolic	Graphitic	Oxidized
N-CNPs	89.65	1.02	9.33	0.11	0.40	0.41	0.08
N-CNPs-NH_3_	88.89	2.43	8.68	0.23	1.14	0.79	0.27

**Table 3:** Comparison of ORR performance of N-CNPs-NH3 with literatures.

Catalysts	Electrolyte	Onset Potential (V)	Main Precursors Materials	Ref.
N-doped carbon	0.1 M KOH	−0.05	Bacillus subtilis	[22]
N-doped nanoporous carbon	0.1 M KOH	0.01	Pomelo peel	[23]
N-doped carbon nanoparticles	0.1 M KOH	−0.02	Sterculia scaphigera	[24]
Oxygen-containing N-doped carbon	0.1 M KOH	−0.04	Glucose and dicyandiamide	[25]
N-CNPs-NH_3_	0.1 M KOH	−0.033	Fresh banana peel	This work
